# Optimising follow up and outcome assessments in traumatic brain injury trials

**DOI:** 10.1186/cc9734

**Published:** 2011-03-11

**Authors:** LJ Murray, JD Cooper, JV Rosenfeld, Y Arabi, A Davies, P D'Urso, T Kossmann, J Ponsford, P Reilly, I Seppelt, R Wolfe, S Vallance, B Howe, M Alkishi

**Affiliations:** 1 The Alfred Hospital, Melbourne, Australia; 2 King Fahad National Guard Hospital, Riyadh, Saudi Arabia; 3 Epworth Health Care, Melbourne, Australia; 4 Monash University, Melbourne, Australia; 5 Adelaide University, Adelaide, Australia; 6 Nepean Hospital, Sydney, Australia

## Introduction

Traumatic brain injury studies predominantly use an assessment of neurological function some time after hospital discharge as the primary endpoint. Recent studies have followed up patients at 6 months after injury with very variable loss to follow up [1,2]. We have established an outcome process that minimises loss to follow up and maximises the quality of the outcome assessment.

## Methods

The DECRA trial is a prospective randomised trial of 155 patients from Australia, New Zealand, and Saudi Arabia. Patients with severe traumatic brain injury and refractory intracranial hypertension were randomly assigned to receive either a decompressive craniectomy or to continue with standard medical management. The primary outcome was patient's neurological function using the Extended Glasgow Outcomes Scale (GOSE) at 6 months after injury. Patients were tracked following hospital discharge by the Research Coordinators at each participating site. The GOSE assessments were conducted by three blinded assessors using structured telephone questionnaires. The assessment team was led by an experienced assessor. Two assessors were located in Australia and one assessor in Saudi Arabia. Assessors were trained using a prepared training package of examples and self-testing exercises. The chief assessor reviewed the outcome assessments performed by the other assessors. Any complex assessments were referred to an assessment panel for a consensus decision.

## Results

DECRA commenced recruitment in 2003 and the last patient was enrolled in April 2010. Research coordinators successfully tracked all surviving patients, which resulted in a 100% follow-up rate for the primary study outcome measure.

## Conclusions

We have successfully completed a prospective randomised controlled trial with zero loss to follow up for the primary outcome measure of GOSE at 6 months. Assessments were reviewed by the chief assessor and a consensus panel if required to ensure consistency of the assessment.

## References

[B1] Bernard SA Ann Surg 2010 252 959 965 10.1097/SLA.0b013e3181efc15f 21107105

[B2] Maas AI Lancet Neurol 2006 5 38 45 10.1016/S1474-4422(05)70253-2 16361021

